# Misdiagnosis of tuberculous infection as pseudo-bursa cyst after total hip arthroplasty: a case report

**DOI:** 10.3389/fsurg.2025.1612055

**Published:** 2025-05-27

**Authors:** Zhen Jia, Zhengqi Chang, Shiyong Wan

**Affiliations:** ^1^ Department of Orthopedics, 961th Hospital of PLA, Qiqihaer, China; ^2^ Department of Orthopedics, 960th Hospital of PLA, Jinan, China

**Keywords:** postoperative tuberculosis infection, pseudo-bursa cyst, total hip arthroplasty, misdiagnosis, case report

## Abstract

This article reports and analyzes a case of postoperative tuberculosis infection in an 80-year-old female after total hip arthroplasty, which was misdiagnosed and mistreated due to imaging findings resembling a pseudo-bursa cyst. The patient had a history of right femoral neck fracture, underwent right total hip arthroplasty 4 years ago, and developed a lump on the posterior side of the right thigh 1 year ago. Initial MRI at another hospital diagnosed it as a pseudo-bursa cyst and underwent excision surgery, but recurred 2 months later. Upon admission, repeated fluid aspiration, biochemical analysis of the fluid (showing high protein, high specific gravity, and positive Rivalta test), PPD, and T-SPOT.TB tests all indicated active tuberculosis infection. Cheese-like necrosis and granuloma formation were found during surgery, confirming postoperative tuberculosis infection. The patient underwent local debridement surgery combined with 9 months of standard HRZE anti-tuberculosis treatment (isoniazid, rifampicin, pyrazinamide, and ethambutol). Follow-up at 9 months showed the lump had disappeared, inflammatory markers returned to normal, and the prosthetic joint remained stable with improved joint function. This case highlights the challenge of tuberculosis infection being masked by common postoperative complications, emphasizing the importance of multidimensional examination and comprehensive diagnosis of diseased tissues to reduce misdiagnosis rates, improve treatment success rates, and enhance patient quality of life.

## Introduction

Hip arthroplasty is an effective treatment for end-stage arthritis and other hip joint diseases, significantly relieving pain, restoring joint function, and improving patients' quality of life. However, postoperative complications, especially infection, are always a concern (1). Traditionally, most postoperative infections are attributed to bacterial pathogens, but in recent years, Mycobacterium tuberculosis infection has received increasing attention due to its insidious course and nonspecific symptoms (2). The spread of joint tuberculosis lesions through blood transmission causing prosthetic joint infection is considered a significant route of transmission, posing a major challenge and affecting 1%–2% of all joint replacement surgeries (3). In the reported cases of tuberculous prosthetic joint infections, hip joint infections account for 53%, while knee joint infections account for 43% (4). Tuberculous infections often present with caseous necrosis and granulomatous reactions, which may resemble pseudo-bursa cysts in some cases, leading to misdiagnosis or missed diagnosis (5). In addition, unlike acute purulent infections, infections caused by Mycobacterium tuberculosis often manifest as chronic low-grade inflammation, with common indicators such as C-reactive protein (CRP) and erythrocyte sedimentation rate (ESR) only slightly elevated, further increasing the diagnostic difficulty (6). This article aims to provide a comprehensive analysis of a case of post-hip arthroplasty tuberculous infection misdiagnosed as a pseudo-bursa cyst, combined with a discussion of relevant domestic and international literature, to provide valuable insights for the diagnosis and treatment of similar cases in clinical practice, and explore how to use multiple diagnostic methods to avoid similar misdiagnoses (7).

## Case present

The patient is an 80-year-old female who underwent a right total hip arthroplasty 4 years ago for a right femoral neck fracture, with no significant discomfort postoperatively. One year ago, a gradually enlarging mass appeared on the posterior-lateral aspect of her right thigh, approximately the size of a fist. The initial MRI showed a cystic lesion with clear borders and thin walls, consistent with the imaging characteristics of a pseudo-bursa cyst (5). The patient underwent excision at another hospital, but the mass recurred and continued to grow. Upon admission to our hospital, a soft mass measuring approximately 15  ×  10 × 10 cm^3^ was palpable on the mid-lateral aspect of the right thigh, with indistinct borders and fluctuance. The mass had normal skin temperature and color, no swelling, and minimal tenderness. The patient's right hip surgical scar had healed well, with normal joint mobility and no significant pain, nerve, or vascular involvement.

During the diagnostic and treatment process, the cystic mass was subjected to 2 ultrasound-guided cyst punctures, with the aspirated fluid being dark brown, turbid, and non-transparent, with no bacterial growth in routine cultures (suggesting a non-purulent infection) (6). Further biochemical testing of the puncture fluid revealed a high protein content of 68 g/L, an elevated specific gravity of 1.048, and a positive Rivalta test, all indicating an exudative nature of the lesion and raising suspicion of tuberculous pus. Concurrent PPD skin test (induration diameter of 18 mm) and T-SPOT.TB testing showed significant positive results, suggesting active tuberculosis infection in the patient (8). Upon admission, the patient's WBC count was 5.86*10^9^/L, RBC count was 4.29*10^12^/L, platelet count was 324*10^9^/L, SC-ALB level was 39.5 g/L, and HGB level was 108 g/L. Additionally, the patient exhibited an elevated ESR of 55 mm/H and a high hs-CRP level of 55.32 μg/ml, suggesting a persistent low-grade inflammatory state. Based on these comprehensive test results and imaging findings showing a double-layered cyst wall with blurred peripheral margins on CT, distinct from typical pseudocystic cysts and possibly containing caseous necrotic components (7).

**Figure 1:**
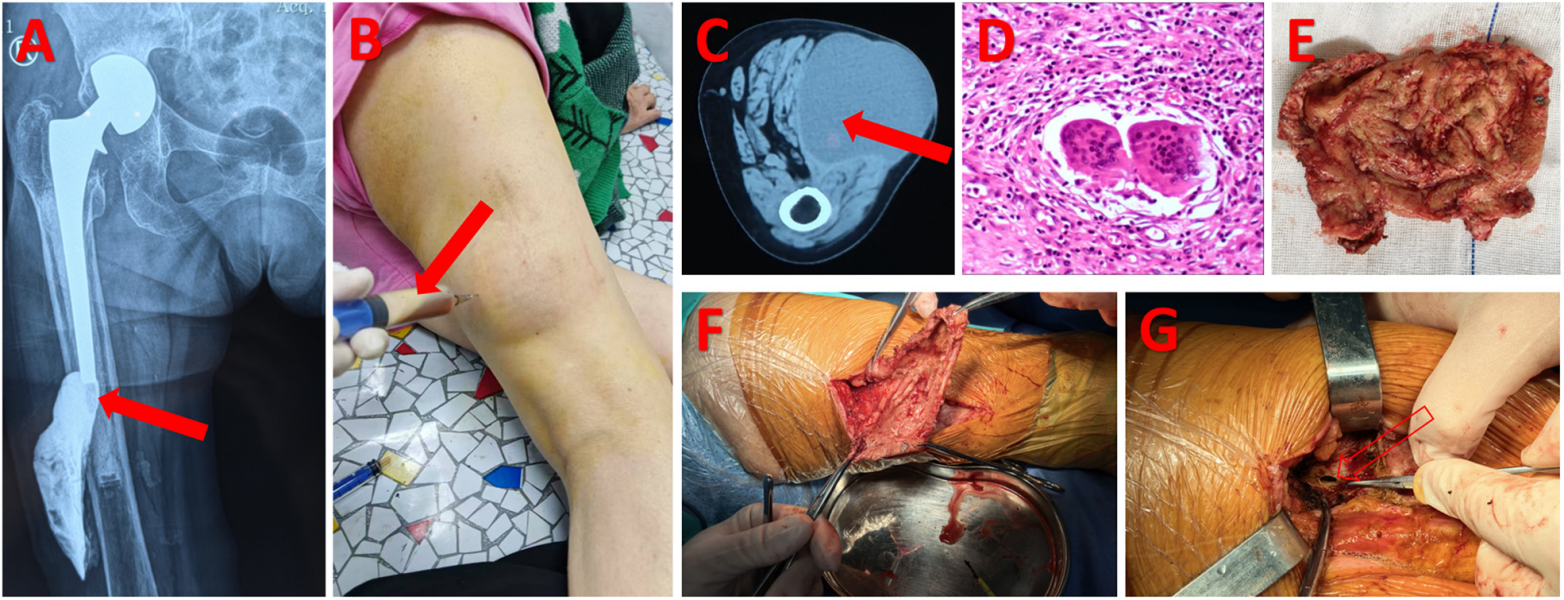
**(A)** Following the administration of contrast medium into the purulent cavity, DR indicates that the abscess cavity may be connected to the implant [indicated by the solid red arrow in **(A)**]. **(B)** Perform a puncture on the liquid within the abscess cavity [indicated by the solid red arrow in **(B)**]. **(C)** A massive abscess cavity is indicated by the CT scan [indicated by the solid red arrow in **(C)**]. **(D)** Postoperative abscess cavity wall pathology suggests inflammatory cell infiltration. **(E,F)** Indicate necrosis resembling cheese seen in the abscess cavity wall during surgery. **(G)** Shows that the abscess cavity was connected to the prosthesis through an oval-shaped defect above the femur during surgery [indicated by the hollow red arrow in **(G)**].

Five days after admission, the patient underwent another local surgical exploration. A longitudinal incision was made on the posterior side of the thigh, and layers of soft tissue were cut open to reveal a complete cyst membrane. A light brown fluid flowed out from inside the cyst, with yellow-brown cheesy tissue seen on the cyst wall. Upon further exploration, a sinus tract was found leading directly to the lateral side of the femur, connecting to the prosthetic joint after hip replacement surgery. Based on intraoperative findings and pathological examination showing inflammatory cell infiltration, cheesy necrosis, and localized granuloma formation, the diagnosis was confirmed as tuberculous infection after hip replacement surgery, rather than a simple pseudobursa cyst. The patient underwent thorough debridement and complete cyst removal surgery, followed by a 9-month standard HRZE (isoniazid, rifampicin, pyrazinamide, ethambutol) anti-tuberculosis treatment regimen, with regular monitoring of inflammatory markers and imaging studies (1) (Figure 1).

## Results

After multiple imaging studies and laboratory tests, the patient's lesion area showed a clear double-layer cyst wall signal, with a blurry outer edge, different from conventional pseudo-bursal cysts. Specifically, CT images showed discontinuous signals in the lesion area, suggesting local tissue necrosis and inflammatory reactions. Fluid biochemical testing results showed a significant increase in protein content (68 g/L), with a specific gravity of 1.048 and a positive Rivalta test, all typical indicators of tuberculous effusion. Additionally, histological examination revealed a large number of lymphocytes and a few macrophages. These comprehensive findings support the diagnosis of tuberculosis infection rather than a simple inflammatory pseudo-bursal cyst (8).

During the 9-month postoperative follow-up period, the patient's local swelling completely disappeared, all inflammatory markers (ESR, CRP, etc.) returned to normal range, and reexamination of imaging showed no fluid reaccumulation or loosening of the artificial joint, with significant improvement in hip joint function. These results fully demonstrate that for cases of cystic lesions after hip arthroplasty, timely identification of tuberculosis infection through multidimensional examination and comprehensive diagnosis can be achieved, leading to targeted treatment plans and avoiding unnecessary surgical interventions or even revision surgeries due to misdiagnosis (9).

## Discussion

In this case, the postoperative imaging findings of the localized cystic lesion in the patient were very similar to a false synovial cyst, which was the main reason for the initial misdiagnosis. False synovial cysts are usually caused by local inflammation stimulating synovial reactions, with a thin and well-defined capsule; while tuberculous infections often present with a double-layered cyst wall structure and irregular signal distribution due to caseous necrosis and granulomatous reactions. The subtle differences in imaging between the two often make it difficult to make an accurate diagnosis based solely on imaging (5). Additionally, the slow growth of tuberculosis bacteria can lead to false-negative results in routine liquid bacterial cultures, further increasing the diagnostic difficulty (6).

The mild elevation of CRP/ESR in patients can be challenging for diagnosis. This is mainly due to immunosenescence in elderly individuals, where both innate and adaptive immune functions decline. This results in weakened T cell responses and impaired monocyte-macrophage functions, leading to reduced release of inflammatory cytokines (such as IL-6, TNF-α) which affects the liver's ability to synthesize acute phase reactant proteins like CRP (10). Additionally, Mycobacterium tuberculosis infection is characterized by granuloma formation, causing chronic focal inflammation that may only trigger local immune responses rather than a systemic acute phase reaction. The extent of CRP and ESR elevation is related to the range of inflammation, with localized infections (such as joint tuberculosis) eliciting weaker systemic responses compared to disseminated tuberculosis (11, 12). However, some clinically significant laboratory tests include high protein content and high specific gravity in liquid biochemical testing, as well as a positive Rivalta test confirming exudate, which are crucial for distinguishing tuberculous pus from other non-tuberculous inflammatory fluids (8). Additionally, PPD skin tests and T-SPOT.TB tests offer strong evidence of active tuberculosis infection, especially for patients with chronic local inflammatory processes but no obvious systemic symptoms (2).

It has been reported in the literature that tuberculosis infections after joint replacement surgery often originate from the reactivation of pre-existing latent lesions or may be newly acquired postoperatively due to changes in the immune environment. Regardless of the scenario, the treatment strategy for tuberculosis infection is drastically different from that of conventional purulent infections (13). While postoperative purulent infections often require revision of the artificial prosthesis, for tuberculosis infections, local debridement combined with long-term anti-tuberculosis drug therapy can effectively preserve the artificial prosthesis and achieve satisfactory results (14). In some cases, single-stage surgery replacement combined with anti-tuberculosis treatment can also be applied to early cases (15). Conversely, for patients with delayed treatment due to misdiagnosis, more extensive surgical debridement and prolonged anti-tuberculosis drug therapy may be necessary to prevent infection spread (16).

In addition, from the perspective of imaging, although CT and MRI have certain value in displaying the range and structure of lesions, the imaging manifestations of tuberculosis infection are diverse due to caseous necrosis and granulomatous reactions, making it difficult to differentiate tuberculosis infection from pseudo-bursa cysts based solely on imaging diagnosis. Therefore, a multidimensional diagnosis is necessary, including needle biopsy, fluid biochemistry and cytology testing, and pathological examination for comprehensive evaluation, which can effectively improve diagnostic accuracy (7).

The experience of this case suggests that when encountering local cystic lesions after hip arthroplasty, one should be highly vigilant about the possibility of tuberculosis infection, especially when the medical history, inflammatory markers, and fluid tests are abnormal. Early recognition and accurate diagnosis are crucial for subsequent treatment, as it can avoid unnecessary excision surgery and instead take targeted debridement and anti-tuberculosis therapy, preserving the prosthetic joint while effectively controlling the infection, thus achieving a good long-term prognosis (9).

## Conclusion

Post-total hip arthroplasty tuberculosis infection can be misdiagnosed due to the similarity in imaging findings with pseudo-bursa cysts. This case was confirmed as tuberculosis infection through detailed biochemical testing of the aspirate, PPD and T-SPOT.TB testing, imaging, and pathology examinations. Local debridement combined with long-term HRZE anti-tuberculosis therapy achieved good treatment outcomes. This case highlights the importance of comprehensive multidimensional examinations for chronic cystic lesions post-hip arthroplasty to prevent misdiagnosis and to develop a treatment strategy that preserves the prosthetic joint as much as possible, improving the patient's quality of life.

## Data Availability

The original contributions presented in the study are included in the article/Supplementary Material, further inquiries can be directed to the corresponding authors.
